# Comparison of Unilateral Knee Arthroplasty with High Tibial Osteotomy in Surgical Treatment of Medial Knee Osteoarthritis

**DOI:** 10.34172/aim.2022.53

**Published:** 2022-05-01

**Authors:** Sinan Zehir, Ercan Şahin

**Affiliations:** ^1^Department of Orthopedics and Traumatology, Faculty of Medicine, Hitit University, Çorum, Turkey; ^2^Department of Orthopedics and Traumatology, Faculty of Medicine, Bülent Ecevit University, Zonguldak, Turkey

**Keywords:** High tibial osteotomy, Osteoarthritis, Unicondylar arthroplasty

## Abstract

**Background:**

High tibial osteotomy (HTO) and unicondylar knee arthroplasty (UKA) are two procedures of choice in moderate medial-sided knee osteoarthritis. In this study, we aimed to compare the outcomes of patients undergoing either unilateral knee arthroplasty or open-wedge HTO both clinically and radiologically.

**Methods:**

Clinical records of 105 patients treated surgically with either medial unilateral knee arthroplasty or high tibial osteotomies were reviewed. Fifty-one cases of HTO (group 1) and 54 cases of unicompartmental knee arthroplasty (group 2) were reviewed. Radiographic follow-up data included Kellgren Lawrence index and mechanical alignment measurements using the PACS system. Clinical and functional follow-up data included range of motion degrees and functional assessment scores (Tegner, Lysholm, Knee Society Score and VAS).

**Results:**

Mean time of follow-up was 66.10±8.14 months and 65.27±6.95 months for groups 1 and 2, respectively. The HTO group had better radiographic improvement and greater range of motion than the unicompartmental knee arthroplasty group. Despite a significant difference in Lysholm Knee Score and a slight difference in VAS score in favor of HTO, both groups were similar with regard to functional outcomes.

**Conclusion:**

Both techniques are satisfactory in terms of functional outcomes about five years after the operation and may be considered in cases of middle-aged medial-sided gonarthrosis (<65 years of age) who do not have additional ligament or compartmental pathology.

## Introduction

 Knee osteoarthritis is one of the leading causes of global orthopedic disabilities with a prevalence ranging from 3.8% to 25%.^1,2^ Likewise, knee arthroplasty has become one of the most common surgical procedures performed in orthopedic practice. With the increasing demands of the elderly in developed countries, more patients with moderate knee osteoarthritis demand pain relief and stay active. Limited benefits of arthroscopic debridement, cartilage repair and autologous chondrocyte implantation in these patients were reported. Unicondylar knee arthroplasty (UKA) to replace medial tibiofemoral articulation and high tibial osteotomy (HTO) to restore altered tibiofemoral axis and shift the mechanical axis to relatively healthy lateral compartment are two procedures of choice for moderate degree medial-sided knee arthroplasty with distinct benefits and issues.^3^

 Despite numerous studies including randomized controlled trials and meta-analyses comparing the results of UKA with HTO, no distinct criteria have been defined to date for patient selection. In these studies, there has been a trend towards performing HTO for relatively young and active patients, and UKA for patients with relatively sedentary habitus, thus making comparison of these techniques problematic.^4^ In this study, we present a single institution’s experience regarding HTO or UKA for surgical treatment of moderate knee osteoarthritis.

## Materials and Methods

 We reviewed patients having Kellgren-Lawrence grade II or higher medial unicompartmental knee osteoarthritis with mild genu varus deformity with complete clinical, radiographic and functional outcome parameters with a follow-up period of at least 5 years. After local ethical board approval (IRB approval ID 208), and obtaining each patient’s informed consent, we started to analyze the patient files from the hospital database. The search of the hospital database revealed that a total of 105 patients underwent surgery for medial-sided knee osteoarthritis using either open wedge HTO (51 patients, mean age: 56.35 ± 3.60) or UKA (54 patients, mean age: 56.90 ± 3.80) from March 2007 until December 2012. Since five HTO patients and four UKA patients were re-operated with total knee arthroplasty, the data of these patients were excluded from evaluation. Preoperative and follow-up full-length weight-bearing radiographs were available for all patients to assess the axial alignment of the weight bearing axis. Patients with lateral or patellofemoral compartmental osteoarthritis (Kellgren-Lawrence score grade II or higher), flexion contracture > 15º, varus malalignment > 10º, severe ligament instability or inflammatory arthropathy were not evaluated. All of the operations were performed by one of several surgeons experienced in knee surgery and the decision to perform either technique was based on the patient’s expectations; HTO might have been preferred over UKA when an individual patient was concerned about any decline in physical functions, although no quantitative limits for either technique have evolved in our practice.

 Although there have been some studies comparing HTO and different methods, no research has compared all of our parameters between HTO and UKA groups. The closest to our study, by van der Woude et al^5^ reported a mean tibiofemoral axis of 6.2 ± 0.3 for HTO group, and 5.8 ± 0.6 for the UKA group. According to this research, effect size was found to be 0.8432740 for comparison of the two groups with similar patient properties. Minimum required sample for each group was found to be 32 at 95% sample power, and 1.6698 critical value.

###  Surgical Procedures

 In the UKA group, institutional adoption of surgical steps was similar to that performed by Vorlat et al^6^ for Oxford^®^ Partial Knee Implant (Biomet, USA). A midline incision was performed, the other two compartments were confirmed to be free of degeneration and the implant was inserted as instructed by the manufacturer. Both tibial and femoral prosthetic components were cemented. Superficial medial collateral ligament was released accordingly. All patients in the UKA group were encouraged for knee motion exercises on the day after surgery and allowed for full weight bearing as tolerated. In the HTO group, after arthroscopic visualization of all joint compartments, a medial opening wedge defect was created and enlarged until the weight-bearing axis was brought through the Fujisawa point.^7^ The defect was filled with allograft and then fixed using a multi-hole plate and cortical screws. Superficial fibers of medial collateral ligament were also released accordingly. All patients in the HTO group were encouraged for knee motion exercises on the day after surgery and allowed for partial weight bearing within 1 week.

###  Outcome Analyses

 Demographic, radiologic and functional evaluation scores were collected including; age, sex, side, preoperative body mass index, pre- and post-operative tibiofemoral angle, pre-operative Kellgren-Lawrence indices according to radiographs, pre- and post-operative knee range of motion measurements and functional evaluation scores (VAS, Lysholm, Tegner and Knee Society scores). On radiological evaluation, the tibiofemoral axis was measured using the PACS software. Varus malalignment was represented as negative (-), whereas valgus axis was given as positive (+).

###  Statistical Analysis

 All statistical analyses were performed using SPSS (SPSS version 18.0 Inc. Chicago, IL. USA). Histograms and analytical methods (Kolmogorov-Smirnov test) were used for determination of normal distribution. Normally distributed data were described with mean (standard deviation), whereas non-normally distributed data were described with median (range). Normally distributed data were compared using independent sample *t* test with Levene homogeneity of variance test and non-normally distributed data were compared using Mann-Whitney test. The results were given as median and min-max. Categorical data were compared using chi-square test or Fisher’s exact test where appropriate. A *P* value less than 0.05 was considered to be statistically significant.

## Results

 BMI and operation duration parameters were significantly higher in the UKA group (*P* < 0.05). Differences in age, gender and side of involvement were statistically insignificant (*P* > 0.05) (Table 1).

**Table 1:** Comparison of Baseline Characteristics and Study Parameters Between the Groups Unmatched and Matched by BMI Score

**Variables**	**Group 1 HTO (n=51)**	**Group 2 UKA (n=54)**	***P*** ** Value**
Age (y), mean ± SD	56.51 ± 3.53	57.13 ± 3.91	0.397^a^
Male gender, n (%)	14 (27.5)	16 (29.6)	0.805^b^
Right sided involvement, n (%)	25 (49.0)	18 (33.3)	0.102^b^
Body-mass index (kg/m^2^), mean ± SD	26.79 ± 4.21	28.97 ± 4.01	0.008^a^
Operation duration, mean ± SD	47.49 ± 7.07	56.11 ± 6.84	0.000^a^

SD, Standard deviation; HTO, High tibial osteotomy; UKA, Unicondylar knee arthroplasty. ^a^Independent samples *t* test. ^b^Chi-square test.

 Regarding the patients undergoing total knee arthroplasty during the follow-up period, one HTO patient had deep wound infection and recovered with local debridement and antibiotics. Two HTO patients had deep venous thrombosis and delayed union was observed in two patients. Two cases of fixation failure, two cases of nonunion and one case of intra-articular fracture were observed in the HTO group during follow-up and underwent revision to total knee arthroplasty. Meniscal bearing dislocation occurred in 4 patients undergoing UKA, all of whom were encountered during early stages of our learning curve and were revised to a total knee arthroplasty within 30 days after the operation. Two UKA patients had superficial infection and two others had synovitis and persistent pain (Table 2).

 The Kellgren-Lawrence score was significantly higher in the HTO group before operation (*P* < 0.05). However, differences in the VAS score, tibiofemoral angle, range of motion, Tegner score, Knee Society Score and Lysholm score were insignificant before operation (*P* > 0.05). After operation, the VAS Score, tibiofemoral angle, range of motion and Lysholm score were significantly higher in the HTO group (*P* < 0.05) (Table 3).

**Table 2:** Postoperative Complications after HTO and UKA

**HTO**	
Deep wound infection	1
Deep venous thrombosis	2
Fixation failure	2
Nonunion	2
Intra-articular fracture	1
**UKA**	
Superficial infection	2
Synovitis	2
Insert dislocation	4

HTO, High tibial osteotomy; UKA, Unicondylar knee arthroplasty.

**Table 3:** Comparison of before and after Operation Outcomes between Patient Groups

**Variables**	**Group 1 HTO (n=51)**	**Group 2 UKA (n=54)**	***P*** ** Value**
Before operation			
Kellgren-Lawrence score mean ± SD	3.22 ± 0.64	2.63 ± 0.78	0.000^a^
VAS score, mean ± SD	7.49 ± 0.88	7.17 ± 1.09	0.158^a^
Tibiofemoral angle, mean ± SD	8.57 ± 2.21	7.83 ± 2.58	0.070^a^
Range of motion, mean ± SD	125.10 ± 7.58	123.30 ± 8.37	0.179^a^
Tegner score, mean ± SD	3.51 ± 0.88	3.44 ± 0.90	0.712^a^
Knee Society Score, mean ± SD	51.39 ± 5.12	52.67 ± 5.27	0.212^b^
Lysholm score, mean ± SD	57.75 ± 6.26	59.02 ± 4.35	0.227^b^
After operation			
Kellgren-Lawrence score mean ± SD	2.69 ± 0.51	2.87 ± 0.55	0.092^a^
VAS score, mean ± SD	3.14 ± 0.57	2.81 ± 0.87	0.010^a^
Tibiofemoral angle, mean ± SD	3.08 ± 1.02	0.94 ± 1.83	0.000^a^
Range of motion, mean ± SD	121.96 ± 6.79	117.04 ± 5.00	0.000^a^
Tegner score, mean ± SD	2.61 ± 0.60	2.56 ± 0.63	0.585^a^
Knee Society Score, mean ± SD	86.49 ± 3.25	87.35 ± 3.97	0.186^a^
Lysolm score, mean ± SD	86.08 ± 3.70	81.98 ± 3.34	0.000^b^

HTO, High tibial osteotomy; UKA, Unicondylar knee arthroplasty. ^a^Mann -Whitney U test. ^b^ Independent samples *t* test.

## Discussion

 Both HTO and UKA have beneficial effects on moderate degree medial-sided gonarthrosis. HTO relieves medial-sided stress. Over the last few decades, there have been several studies on quantitative and functional outcomes after UKA and HTO and many comparative studies are available in the literature.^4,8^ In our study, we compared the radiographic and clinical outcomes of a matched cohort of medial-sided gonarthrosis patients treated operatively by HTO and UKA with a minimum follow-up of 5 years retrospectively.

 UKA with Oxford^®^ Partial Knee Implant has been available for about two decades with 10-year survival being reported to be as high as 84%. Its use was advised as a first-choice treatment method in medial unicompartmental arthritis, since it well preserves the bone stock and allows an easy revision to a total replacement, when necessary.^6^

 HTO, either with opening or closing wedge technique, is a widely performed treatment choice in mild to moderate degree unicompartmental knee osteoarthritis. We prefer the opening wedge technique basically for benefits such as avoidance of fibular osteotomy and preservation of patellar tendon length. Success of HTO is dependent on appropriate patient selection such as young patients (< 60 years of age) without significant flexion contracture and ligament instability.^9^

 The impact of correction of tibiofemoral alignment on clinical outcome is still controversial.^4^ It was shown that inadequate alignment following HTO is associated with poor outcomes.^10^ However, in general, it is accepted that 3º to 6º valgus should be aimed to achieve satisfactory results following HTO.^11^ We measured a median correction of 6° [2°/ + 11°] and a median tibiofemoral angle of 3°[0°/ + 5°] at the end of follow-up of HTO patients. Actually, these correction values are “remaining correction” after five years of follow-up. The cartilage loss on the medial compartment during this period and eventual loss of correction is not taken into consideration. This effect may explain the differences in tibiofemoral axis values of our study with previously reported values.

 Regarding functional assessment, we achieved similar satisfactory outcomes between two groups with regard to functional outcomes like Knee Society Scores and Tegner activity scores, despite a significant difference in Lysholm scores at the latest follow-up which do not seem to be of clinical importance. Yim et al compared HTO and UKA in terms of clinical outcomes and return to recreational activities. In line with our findings, they reported that there were no significant differences between the two groups in Tegner activity scores and return to recreational activities.^12^ Börjessön et al reported in their prospective follow-up study that outcome scores were similar in patients receiving either HTO or UKA.^13^ A meta-analysis performed by Spahn et al compared the results of studies comparing HTO vs. UKA. They reported that although UKA performed better in the first 12 years post-operatively, no difference existed afterwards. They also conclude that HTO is more suitable for younger patients with high activity demands and UKA for older patients demanding pain relief accepting activity restriction.^8^ In another meta-analysis, it was reported that although HTO revealed better range of motion than UKA, no difference in a specific knee score could be demonstrated.^4^ The results of the studies and meta-analyses can be interpreted as no major advantage for either technique over the other. In our study, we observed a significant difference in only one (Lysholm) of the two knee outcome scoring systems. There was an obvious increase in both scores in both groups (between 25–30 points approximately) when compared to baseline scores. These results are also comparable with the results of previous studies.

 In a study by Börjessön et al, pain during ambulation was investigated, reporting decreased pain in both groups without any significant difference.^13^ In our study, we found a slightly better decrease in VAS scores in HTO patients, compared to UKA patients. This difference may be contributed to the relatively shorter follow-up period.

 At 5 years of follow-up, we observed a survival rate of 90.2% in the HTO group and 92.6% in the UKA group. This finding is comparable with previous reports.^8^ It should be kept in mind that revision of both HTO and UKA may be challenging. Previous incision and altered joint biomechanics in HTO patients and need for augmentation blocks or stems in UKA revisions may further complicate long-term TKA outcomes in these groups of patients.^14^ We believe that with better patient selection and improvement of surgical technique by reaching the plateau of the learning curve, the long-term survival rates may be improved

 Our study has several limitations. First, this study was based on archive registry data. Even in the most sophisticated and well-structured registration systems, selection bias remains a potential confounding factor. In our study, although being quite few, we excluded cases with missing data. Another limitation is the retrospective nature of our study design. Although we used objective statistical tools for matching two sets of data, this analysis can never be as strong as the data provided by a prospective-randomized trial.

 Both HTO and UKA are satisfactory in terms of functional outcomes about five years after operation with more than 90% survival. Both techniques may be considered in young patients (< 65 years of age) with moderate degree medial-sided gonarthrosis patients without additional ligament or compartmental pathology.
